# In Vitro Evaluation of Manual, Electric, and Sonic Toothbrushing on Composite Restoration Surface Properties and Retention in Primary Teeth

**DOI:** 10.3390/dj14090583

**Published:** 2026-09-10

**Authors:** Francesco Saverio Ludovichetti, Simone Smania, Ernesto Comitale, Marco Rossi, Gabriel Leonardo Magrin, Edoardo Stellini, Sergio Mazzoleni

**Affiliations:** 1 Department of Neurosciences, Dentistry Section, Padova University, 35122 Padova, Italy; simone.smania@studenti.unipd.it (S.S.); ernesto.comitale@unipd.it (E.C.); edoardo.stellini@unipd.it (E.S.); sergio.mazzoleni@unipd.it (S.M.); 2 Vittorio Veneto Hospital, 31029 Vittorio Veneto, Italy; marco.rossi@aulss2.veneto.it; 3 Department of Dentistry, Federal University of Santa Catarina—UFSC, Florianópolis 88040-535, SC, Brazil; gabriel.magrin@ufisc.br

**Keywords:** primary teeth, powered toothbrushes, composite restorations, surface roughness, dislodgement load

## Abstract

**Background:** The longevity of composite restorations in primary teeth depends on the preservation of both mechanical performance and surface integrity under daily oral hygiene procedures. While powered toothbrushes have been widely investigated for plaque control, their potential effects on restorative materials in the primary dentition remain insufficiently characterized. This preliminary in vitro study evaluated the influence of manual, oscillating-rotating electric, and sonic toothbrushing on the surface microhardness, surface roughness, and shear resistance of resin composite restorations in primary teeth. **Methods:** Thirty extracted non-carious primary teeth were standardized, cavity-prepared, and restored using a self-etch adhesive system and a resin composite material. Specimens were randomly assigned to three groups (n = 10) according to brushing modality: manual, electric, or sonic. After a standardized 60 min brushing simulation under controlled conditions, surface microhardness was measured using a Vickers microhardness tester, surface roughness was assessed with contact profilometry, and restoration retention was evaluated as maximum load at failure under shear-type mechanical loading. Surface morphology was further analyzed by scanning electron microscopy. **Results:** No statistically significant differences were observed among groups in surface microhardness (*p* = 0.606) or maximum dislodgement load (*p* = 0.806). In contrast, surface roughness differed significantly according to toothbrush type (*p* < 0.001), with the highest Ra values recorded in the sonic group. Scanning electron microscopy corroborated these findings, showing progressively greater surface irregularities, porosities, and microdefects from manual to sonic brushing. Under the present in vitro conditions, the sonic toothbrush group showed the highest final surface roughness values, whereas no significant differences were observed in microhardness or restoration retention. **Conclusions:** Under the present short-term in vitro conditions, the sonic toothbrush group showed higher final surface roughness values than the manual and electric groups, while no significant differences were observed in microhardness or restoration retention. These results are limited to the specific toothbrush systems tested and these findings should be interpreted as preliminary in vitro results from an exploratory sample with limited statistical power.

## 1. Introduction

Dental caries remains highly prevalent in children, and resin composites are widely used for restoring primary teeth. Although various restorative materials are available, there is limited evidence on the optimal choice for primary dentition. In pediatric dentistry, various restorative material options are available (CR, GIC and RMGIC) [1,2]. Currently, glass-ionomer cements and resin composites are the most used restorative materials in dentistry. Their selection is based not only on their physical and chemical properties but also on the specific dental care needs of the child, including the extent of tooth destruction, the functional and aesthetic consequences of caries, and the child’s behavior [3]. Although placing restorations is a common treatment approach in clinical practice, there is insufficient scientific evidence to determine the best filling material for treating caries in primary teeth [4]. Worldwide, resin composite combined with adhesive systems is the material of choice for restoring primary teeth in approximately 25% of cases [5,6]. This material shows satisfactory effectiveness when used with local anesthesia and rubber dam, regardless of the brand of resin composite, with a success rate of around 90% on occlusal and occlusoproximal surfaces of primary teeth [2]. Regarding the adhesive system, a systematic review suggests that ‘etch-and-rinse’ adhesives perform better in primary teeth compared to ‘self-etch’ systems [7]. One of the proposals to improve the bond stability of restorations placed in primary teeth is shortening the etching time in dentin for etch-and-rinse adhesive systems. This protocol is based on good results of in vitro studies [8].

Compared with glass-ionomer cements, resin composites generally exhibit higher mechanical performance and surface hardness [9].

The clinical performance of composite restorations depends on their surface properties, including hardness and roughness. Surface roughness, in particular, can increase over time due to mechanical abrasion from toothbrushing and may influence plaque accumulation and restoration longevity. The development of advanced restorative materials has introduced several clinical advantages. For example, bulk-fill composite resins can be applied in increments of up to 4 mm, exceeding the 2 mm incremental limit typically recommended for conventional composites. This allows clinicians to bypass the incremental layering technique while still ensuring sufficient depth of cure and polymerization. [10]. When selecting an appropriate restorative material, several factors must be considered, particularly its mechanical and physical properties. Key parameters to consider include the depth of polymerization, surface hardness, surface roughness, and the material’s resistance to acidic degradation and mechanical wear [11]. Polymerization depth is defined as the maximum thickness of resin that can be effectively converted from monomer to polymer. This parameter is influenced by several factors, including the type of resin, the filler content, the exposure time, the intensity and wavelength of the curing light, and the distance between the light source and the material surface in photopolymerizable systems [12]. Hardness refers to a material’s resistance to permanent deformation or indentation when subjected to a mechanical force exerted by a harder object [13]. Despite the widespread use of powered toothbrushes, their effects on the surface properties and retention of composite restorations in primary teeth remain insufficiently characterized. Most evidence on toothbrush-induced surface changes relates to permanent dentition or enamel/dentin substrates, with limited data on composite restorations in deciduous teeth. Therefore, the aim of this in vitro study was to evaluate the effect of manual, electric, and sonic toothbrushing on selected surface properties and retention of composite restorations in primary teeth under standardized laboratory conditions. The null hypothesis was that there would be no difference among the three toothbrushing modalities (manual, electric, and sonic) in terms of surface microhardness, surface roughness, and restoration retention.

## 2. Materials and Methods

Thirty non-carious primary teeth scheduled for extraction in two private dental clinics due to physiological root resorption or for orthodontic purposes were included in the present study, comprising 12 canines and 18 molars. The duration of storage ranged from 1 day to 4 weeks, and all experimental procedures were completed within 8 weeks from extraction.

To reduce specimen heterogeneity, group allocation was performed using stratified randomization by tooth type. Specifically, canines and molars were first separated into two strata, and within each stratum, specimens were randomly assigned to the three experimental groups to obtain the same distribution of tooth types in each group, namely 4 canines and 6 molars per group. Randomization was performed using a computer-generated random sequence. Only teeth with an intact buccal surface large enough to allow preparation of a standardized Class V cavity were included. Teeth with visible cracks, carious lesions, extensive restorations, or severe root resorption were excluded. Although both primary canines and molars were included, their distribution was balanced across groups, which likely reduced the influence of tooth-type heterogeneity on the comparisons. The extracted teeth were stored in distilled water at room temperature until further use, no chemical disinfection procedure was performed to avoid potential alteration of the dental substrate. Specimens were inspected before cavity preparation, and any teeth showing surface defects or damage were excluded from the study.

Prior to cavity preparation, all specimens were subjected to surface standardization. This study was designed as a preliminary pilot in vitro investigation aimed at exploring the effect of different toothbrushing modalities under standardized laboratory conditions. Due to the exploratory nature of the study and the limited availability of extracted sound primary teeth meeting the inclusion criteria, 30 specimens were included and equally allocated among the three experimental groups.

Each tooth was polished for 30 s using a rotary polishing brush mounted on a micromotor operating at a constant speed (40,000 rpm) to achieve a uniform baseline condition of the dental surfaces. Standardized Class V cavity preparations were performed on the buccal surface of all specimens using the same spherical diamond bur mounted on a high-speed handpiece under continuous water cooling. Cavities were located approximately 1 mm above the cementoenamel junction and standardized to 3 mm in mesiodistal length, 2 mm in occlusocervical height, and 2 mm in depth. Dimensions were checked using a periodontal probe to ensure consistency among specimens. All cavity preparations were performed by the same operator, and the bur was replaced at regular intervals to ensure consistent cutting efficiency [14]. After cavity preparation, all cavities were restored following the manufacturer’s instructions for the self-etch adhesive system Tokuyama Bond Force II (Tokuyama Dental, Tokyo, Japan). Enamel and dentin surfaces were gently cleaned and dried, and the adhesive was applied using a microbrush for 30 s and then light-cured for 30 s. No separate phosphoric acid etching step was performed. The curing unit emits in the blue light spectrum (wavelength range approximately 440–480 nm). The curing tip was positioned at a distance of less than 1 mm from the restoration surface, and the light output was verified before each session using the internal radiometer of the curing unit. with a paste composite resin (Tokuyama Omnichroma, Tokuyama Dental, Tokyo, Japan) in a single increment and light-cured for 30 s using a LED curing unit (3M Elipar DeepCure-L, 3M ESPE, Seefeld, Germany). The light intensity was verified before each session using the internal radiometer of the curing unit and was approximately 1470 mW/cm^2^. The curing tip was positioned as close as possible to the restoration surface, perpendicular to the cavity, during the entire curing cycle. After polymerization, all restorations were finished and polished using a standardized protocol. Finishing was performed with fine and extra-fine diamond finishing burs (Diatech HP burs, Diatech, Geneva, Switzerland) under water cooling to remove excess material and standardize the restoration contour. Polishing was then carried out using a sequential disc-based polishing system (Sof-Lex Pop-On, 3M ESPE, Saint Paul, MN, USA), following the manufacturer’s recommended grit sequence (extra-fine, fine, medium, coarse). Each disc was used at a rotational speed of approximately 15,000 rpm for 10 s per surface, under light manual pressure, for a standardized total polishing time of 60 s per specimen. All finishing and polishing procedures were performed by the same operator under consistent conditions before the toothbrushing simulation. All finishing and polishing procedures were performed by the same operator under consistent conditions before the toothbrushing simulation. After restoration, the specimens were randomly divided into three experimental groups (n = 10 per group) according to the type of toothbrush used:

- Group 1: manual toothbrush (Elmex Interx).
- Group 2: electric toothbrush (Oral B iO).
- Group 3: sonic toothbrush (Curaspet Sonic).

All restored specimens were subjected to a standardized toothbrushing procedureaccording to group allocation (manual, electric, sonic) for a duration of 60 min, with specimen hydration maintained throughout the procedure. All restored specimens were subjected to a standardized toothbrushing procedure according to group allocation (manual, electric, sonic) for a duration of 60 min, with specimen hydration maintained throughout the procedure. The toothbrushes used were:

- Elmex Interx (manual, soft bristles; brush head model: Interx Compact Soft, GABA International, Therwil, Switzerland);
- Oral-B iO (oscillating-rotating electric toothbrush, regular cleaning mode; brush head model: iO Ultimate Clean, Oral-B, Procter & Gamble, Schwalbach am Taunus, Germany), operating at approximately 10,500 oscillations and 8800 rotations per minute according to the manufacturer’s specifications;
- Curaspet Sonic (sonic toothbrush, standard mode; brush head model: Curaspet Sonic Soft, Curaprox, Kriens, Switzerland), operating at a frequency of approximately 31,000 vibrations per minute.

Brush heads were replaced for every specimen to minimize the effect of bristle wear on abrasiveness.

For the manual group, brushing was performed using standardized back-and-forth movements at a frequency of approximately 2 Hz. For the powered toothbrushes, the manufacturers’ recommended operating modes were used, with the brush head held in light contact with the restoration surface under the calibrated load. Specimen hydration was maintained using distilled water at room temperature; no toothpaste or abrasive slurry was used during the brushing protocol. This represents a limitation of the experimental model, as the absence of dentifrice does not fully reproduce clinical brushing conditions. Brushing force was objectively standardized at 2 N for all experimental groups, in accordance with previous in vitro toothbrushing abrasion protocols. The force was calibrated before each session using a precision balance/load-control system, and the toothbrush head was applied perpendicular to the restoration surface under controlled contact conditions. All brushing procedures were performed by a single operator following the same standardized protocol for specimen positioning, angulation, and brushing movement [15]. Each specimen was fixed in a stable position during brushing to maintain a consistent contact pattern between the bristles and the restoration surface. The applied load was periodically checked to ensure that the target force was maintained throughout the procedure. Following the toothbrushing simulation, all restored specimens were subjected to three quantitative analyses: surface microhardness, surface roughness, and maximum load at failure under shear-type loading. All measurements were performed by a single trained operator to minimize inter-operator variability, and testing conditions were standardized to ensure reproducibility.

Surface microhardness was evaluated using a Vickers diamond indenter (Vickers microhardness tester, Shimadzu, Kyoto, Japan) under a test force of 10 N (approximately 1 kgf) and a dwell time of 20 s. Indentations were performed on the central area of each restoration to avoid marginal effects and potential influence from surrounding enamel or dentin. For each specimen, five indentations were placed at non-overlapping sites, and the mean value was used for statistical analysis. Hardness values are reported as Vickers hardness (HV1) and were calculated automatically by the instrument software according to the standard Vickers equation:

$$HV=0.1891\cdot \frac{F}{d^{2}}$$

where F is the test force in Newtons and d is the arithmetic mean of the two diagonal measurements of the indentation in millimeters [16].

**Figure 1:**
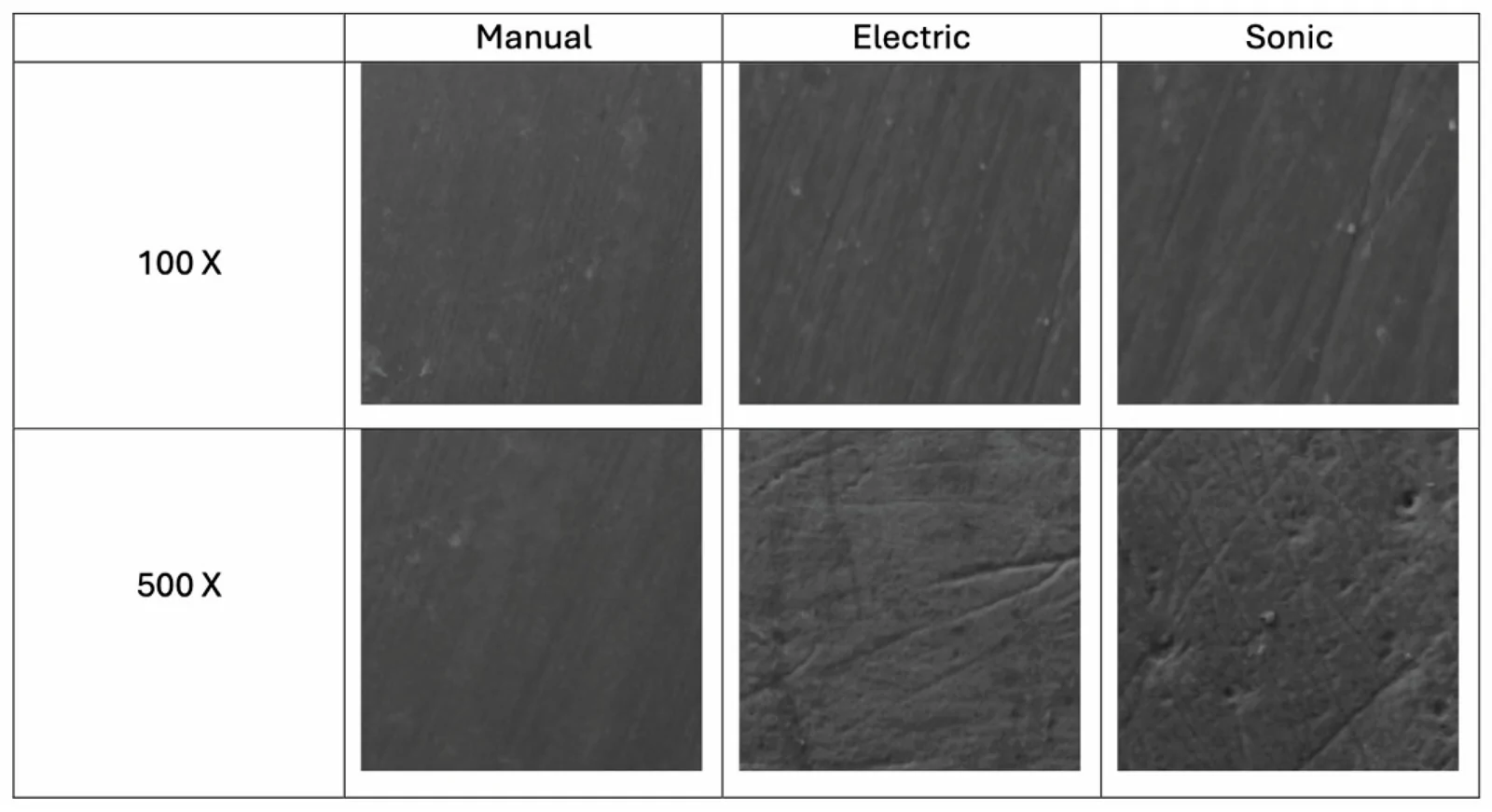
Scanning electron microscopy (SEM) images of composite surfaces after toothbrushing with different toothbrush types. The upper row shows representative surfaces at 100× magnification, while the lower row shows the same groups at 500×. From left to right: manual toothbrush, electric toothbrush, and sonic toothbrush. A progressive increase in surface roughness, porosity, and microdefects is visible from manual to sonic brushing, in agreement with the profilometric roughness data obtained in the present in vitro model. These SEM observations are qualitative and illustrative of surface morphology, and were not used for quantitative analysis.

Surface roughness was measured using a contact profilometer (Surftest SJ-210, Mitutoyo Corporation, Kanagawa, Japan). Each specimen was placed on a stable, vibration-free platform, and measurements were performed in a controlled laboratory environment. For each restoration, three consecutive linear scans were performed across the central region of the restored surface. The profilometer stylus traversed a total distance of 4.0 mm, but the Ra values were calculated over a 2.0 mm evaluation length centered on the composite restoration, ensuring that the evaluation segment remained within the restorative material and avoided the restoration margins and the enamel–composite interface. The cut-off length was 0.8 mm and the stylus speed was 0.5 mm/s. The diamond-tipped stylus had a radius of 5 um and applied a constant vertical force of 0.75 mN. The arithmetic mean roughness (Ra, expressed in um) was calculated as the mean of the three measurements, in accordance with ISO 4288 (ISO 4288:1996; Geometrical Product Specifications (GPS)—Surface texture: Profile method—Rules and procedures for the assessment of surface texture. International Organization for Standardization: Geneva, Switzerland, 1996). After profilometric measurements, morphological characteristics of the samples were examined using a scanning electron microscope (SEM). Since non-conductive samples require a conductive coating for proper imaging, all specimens were sputter-coated with a gold layer of approximately 20 nm prior to observation. The instrument used was a Cambridge Stereoscan 440 (Leica Microsystems s.r.l., Milan, Italy), equipped with a Philips PV9800 EDS microanalysis system (Leica Microsystems s.r.l., Milan, Italy), available at the Industrial Engineering Department of the University of Padua. Images were acquired using the secondary electron detector. For each experimental group, three specimens were randomly selected for SEM analysis. For each specimen, two to three representative fields were examined at 100× and 500× magnification, focusing on the central area of the restoration. The images shown in Figure 1 were selected as representative of the typical surface morphology observed in each group. No quantitative SEM analysis was performed; SEM findings are therefore reported as qualitative morphological observations supporting the profilometric roughness data.

Restoration retention was assessed as the maximum load at failure under a shear-type mechanical challenge. After toothbrushing simulation and 24 h of storage in distilled water at 37 °C, each specimen was embedded in self-curing acrylic resin (Ortho-Jet, Lang Dental Mfg. Co., Wheeling, IL, USA) using cylindrical molds, with the restored buccal surface oriented upward and accessible for loading. A universal testing machine (Instron 3345, Instron Corp., Norwood, MA, USA) was used to apply a controlled load at a constant crosshead speed of 1.0 mm/min through a chisel-shaped stainless-steel loading head. The loading head was positioned on the occlusal/incisal edge of the restoration, parallel to the restoration–tooth interface, and as close as possible to this interface without directly contacting the marginal ridge. Specimen positioning was standardized using a custom jig to ensure consistent orientation and loading point across all specimens. The load was applied until failure occurred, and the maximum force required to dislodge the restoration from the tooth surface was recorded in Newtons (N). Data acquisition was performed using Bluehill Universal software V 4.25 (Instron Corp., Norwood, MA, USA) [17].

### Statistical Analysis

Statistical analysis was performed to test the null hypothesis of no difference among the three toothbrush groups (manual, electric, sonic) for each outcome variable. Continuous data (surface hardness, surface roughness Ra, maximum dislodgement load) were summarized using mean, standard deviation, median, and range. Data distribution within each group was checked with the Shapiro–Wilk test, and homogeneity of variances was assessed with Levene’s test. All outcome variables met the assumptions of normality and homogeneity of variances required for one-way ANOVA. Accordingly, one-way ANOVA was applied, followed by Tukey’s HSD post hoc test for pairwise comparisons when the global F-test was significant (α = 0.05). Groups sharing the same uppercase letter in the tables were considered not significantly different, whereas different letters indicated statistically significant differences. For ANOVA models, exact *p*-values and effect sizes (η^2^, eta-squared, representing the proportion of total variance explained by the factor) were reported. For significant post hoc comparisons, Tukey HSD results were presented with mean differences and 95% confidence intervals. All analyses were performed using SPSS version 28.0 (IBM Corp., Armonk, NY, USA).

## 3. Results

- Surface hardness. Mean Vickers microhardness (HV1) values were comparable across groups (Manual: 80.36 ± 2.93; Electric: 80.45 ± 3.79; Sonic: 81.86 ± 4.34; Table 1). A one-way ANOVA detected no statistically significant difference among groups $\left(F_{2,27}=0.51,\;\;p=0.606;\;\;\eta^{2}=0.036\right)$, indicating a small effect size.
- Surface roughness. All specimens underwent the same finishing and polishing protocol before toothbrushing in order to standardize the initial restoration surface. Surface roughness (Ra, micrometers) differed significantly among groups (Figure 1), with the highest values recorded after sonic brushing (Manual: 0.2298 ± 0.0244; Electric: 0.2835 ± 0.0328; Sonic: 0.3383 ± 0.0310; Table 2). One-way ANOVA confirmed a significant effect of toothbrush type on surface roughness $\left(F_{2,27}=33.47,\;\;p<0.001;\;\;\eta^{2}=0.713\right)$, indicating a large effect size. Tukey HSD post hoc analysis showed significant differences between manual and electric brushing $\left(mean\;difference=0.0537\;\mu m,\;\;95\%CI=0.0265\;to\;0.0809,\;\;p=0.001\right)$, between manual and sonic brushing $\left(mean\;difference=0.1085\;\mu m,\;\;95\%CI=0.0813\;to\;0.1357,\;\;p=0.001\right)$, and between electric and sonic brushing $\left(mean\;difference=0.0548\;\mu m,\;\;95\%CI=0.0276\;to\;0.0820,\;\;p=0.0010\right)$.
- Restoration retention under shear load. The maximum dislodgement load (N) was comparable across groups (Manual: 154.58 ± 20.56; Electric: 159.54 ± 25.35; Sonic: 153.88 ± 15.94; Table 3). No statistically significant differences were detected among groups $\left(F_{2,27}=0.217,\;\;p=0.806;\;\;\eta^{2}=0.016\right)$, indicating a small effect size.

## 4. Discussion

Despite the vast commercial availability of adhesive restorative systems, the majority of contemporary formulations are engineered specifically for permanent dentition. Consequently, the restorative requirements of primary teeth have remained significantly under-addressed. The clinical management of primary teeth is inherently complex due to several histomorphological factors, including accelerated caries progression, reduced enamel thickness, distinct pulpal morphology, and limited crown dimensions. Achieving a robust interfacial bond to both primary enamel and dentin is therefore paramount, as it facilitates the preservation of dental tissue while ensuring the esthetic and functional longevity of the restoration [18].

Toothbrushing remains the primary method for maintaining oral hygiene at home. Its role in disrupting plaque biofilm and promoting gingival health is indispensable. Manual toothbrushes are the most widely used worldwide due to their affordability, availability, and ease of use, and their effectiveness varies depending on technique, duration, and individual dexterity [19]. To address the limitations of manual brushing, powered toothbrushes—both electric and sonic—have been developed to improve plaque removal and user compliance. These devices aim to standardize brushing performance and reduce reliance on brushing technique. Electric toothbrushes, with their small round heads, provide effective mechanical plaque disruption [20]. Sonic toothbrushes, on the other hand, use high-frequency vibrations to create fluid dynamics that help remove plaque even in hard-to-reach areas, improving biofilm disruption. Ultrasonic and multidirectional brushes combine mechanical movement with ultrasonic frequencies to further enhance cleaning efficacy [21].

Hardness is commonly defined as a material’s resistance to deformation. However, hardness is not an intrinsic property, as the hardness value of a material results from a specific measurement procedure. Vickers microhardness is a well-established and suitable method for investigating the properties of human hard dental tissues as well as dental restorative materials [22]. The results of the present study did not reveal statistically significant differences in surface hardness. Within the limitations of this study, the different toothbrushing technologies investigated did not significantly affect surface hardness.

The surface roughness plays a clinically relevant role in the longevity of restorations. A roughened surface may act as a nidus for plaque accumulation, promoting biofilm maturation and secondary caries formation [23]. It may also compromise the aesthetic quality and stain resistance of the restoration over time. Surfaces with a roughness value (Ra) above 0.2 micrometers have been shown to retain significantly more plaque and are harder to clean effectively [24]. In our study, all experimental groups showed measurable surface roughness after the brushing protocol, with the highest Ra values observed in the sonic group, as confirmed both by profilometric data and SEM. However, the three toothbrush systems tested (Elmex Interx, Oral-B iO, and Curaspet Sonic) differ not only in their mode of action (manual, oscillating-rotating electric, and sonic) but also in brush-head geometry, filament characteristics, stiffness, and movement frequency. Therefore, the observed differences in surface roughness cannot be attributed exclusively to the general brushing technology and should be interpreted as specific to the toothbrush systems evaluated in this study.

Although the differences in surface roughness among toothbrush types were statistically significant, their clinical relevance must be interpreted with caution. The brushing duration in this in vitro model represents a short-term challenge compared with cumulative brushing over months or years in real-world conditions. 

Testing the maximum load at failure under shear-type loading is frequently employed to evaluate the retention of restorative materials to the tooth structure [25]. A possible advantage of testing maximum dislodgement load under shear-type loading is that this method is easy to perform compared to microtensile testing of restorative materials [26]. Moreover, shear-type loading is more representative of the clinical forces to which these restorations are subjected [27]. In our study, no statistically significant differences were observed regarding maximum dislodgement load. This suggests that, in the short term, the adhesive strength of the restorations is not compromised despite the use of different toothbrushing technologies. However, the cumulative effects of mechanical wear, particularly those associated with high-frequency toothbrushing, may not be fully represented by a single-cycle debonding test. Repeated brushing over time may induce microdamage at the adhesive interface, potentially leading to a gradual reduction in interfacial strength or marginal deterioration, which could ultimately contribute to restoration failure in the long term.

The present investigation should be regarded as a preliminary laboratory evaluation of the effect of different toothbrushing modalities on selected surface and retention-related properties of composite restorations in primary teeth. The present in vitro study aimed to investigate whether different brushing technologies—manual, electric, and sonic—affect the surface hardness, surface roughness, and shear bond strength of resin composite restorations placed in primary teeth. Although no statistically significant differences were found in bond strength and surface hardness among the groups, significant differences were observed in surface roughness values, with sonic toothbrushes producing the most pronounced alterations. Our results indicate that, under controlled laboratory conditions, brushing with a sonic toothbrush result in a significantly higher surface roughness compared to both manual and electric brushing. The study design was intended as a cross-sectional end-point laboratory comparison among three toothbrushing modalities rather than a longitudinal within-specimen assessment. Future studies should include either unbrushed controls or baseline-to-final repeated measurements to better quantify brushing-induced surface changes. Although the present model allowed a standardized comparison among different toothbrushing modalities, it does not reproduce the full complexity of the oral environment, including saliva, biofilm, dietary factors, pH fluctuations, occlusal loading, cumulative wear, and interindividual variability in brushing habits. Therefore, the present findings should not be directly extrapolated to the clinical longevity or degradation behavior of restorations in primary teeth. A major Ation of the present study is the short duration of the brushing protocol. Although the 60 min exposure allowed a standardized comparison of the effects of manual, electric, and sonic toothbrushing, it cannot reproduce the cumulative mechanical wear associated with repeated daily brushing over months or years. Therefore, the present findings should be interpreted as short-term in vitro data rather than as evidence of long-term material degradation. The study was intentionally designed as a preliminary laboratory evaluation focused on the immediate effects of different brushing modalities under standardized conditions. Another limitation of the present study is the relatively small sample size and the absence of an a priori power analysis. Therefore, the lack of significant differences observed for microhardness and restoration retention should be interpreted with caution, as these findings may reflect either a true absence of effect or limited statistical power. For this reason, the present results should be regarded as preliminary and hypothesis-generating rather than definitive. Future studies including extended cyclic brushing and/or additional aging procedures are needed to better simulate clinically relevant long-term exposure. A further limitation of the present study is the absence of an unbrushed control group and the lack of baseline surface roughness measurements before toothbrushing. Therefore, the present results should be regarded as preliminary and hypothesis-generating rather than definitive. Furthermore, failure modes after mechanical testing were not assessed, which limits the interpretation of the retention results in terms of adhesive versus cohesive failure.

In addition, although the profilometer stylus traversed a total distance of 4.0 mm, the Ra values were calculated over a 2.0 mm evaluation length centered on the composite restoration, ensuring that the evaluation segment remained within the restorative material and avoided the restoration margins and the enamel–composite interface. Therefore, the reported Ra values represent final post-brushing surface conditions and cannot be interpreted as true changes from baseline $\left(\Delta Ra\right)$. Therefore, the present analysis allows comparison among brushing modalities under standardized final experimental conditions, but it does not allow quantification of the absolute contribution of brushing relative to the initial post-polishing surface state.

## 5. Conclusions

Within the limitations of this in vitro short-term study, after a standardized 60 min brushing protocol, the sonic toothbrush group showed higher final surface roughness values of composite restorations in primary teeth than the manual and electric groups, whereas no significant differences were observed in microhardness or restoration retention among the tested groups. These findings apply to the specific toothbrush models tested and should not be generalized to all manual, electric, or sonic toothbrushes. Moreover, they should be interpreted as preliminary laboratory data and do not demonstrate equivalence between groups for microhardness or restoration retention. These findings should be interpreted as preliminary laboratory data and do not demonstrate clinical degradation or long-term clinical risk. Further studies under more clinically relevant conditions are needed to clarify the possible clinical implications.

## Figures and Tables

**Table 1:** Vickers microhardness (HV1) by group $\left(n=10\right)$. One-way ANOVA: $F_{2,27}=0.51$*,* p=0.606, $\eta^{2}=0.036$. Different uppercase letters indicate significant differences in post hoc comparisons; groups sharing the same letter are not significantly different.

Group	Mean ± SD	Median	Min–Max	Statistical Results
Manual	80.36 ± 2.93	80.45	76.5–83.2	A
Electric	80.45 ± 3.79	80.6	74.0–85.0	A
Sonic	81.86 ± 4.34	82.0	75.0–88.1	A

**Table 2:** Surface roughness (*Ra*, μm) by group $\left(n=10\right)$. One-way ANOVA: $F_{2,27}=33.47$*,* p<0.001, $\eta^{2}=0.713$. Tukey HSD showed significant differences among all groups. Different uppercase letters indicate statistically significant differences; groups not sharing the same letter differ significantly.

Group	Mean ± SD	Median	Min–Max	Statistical Results
Manual	0.2298 ± 0.0244	0.2241	0.1941–0.2718	A
Electric	0.2835 ± 0.0328	0.2828	0.2336–0.3400	B
Sonic	0.3383 ± 0.0310	0.3430	0.2820–0.3875	C

**Table 3:** Dislodgement load (N) by group $\left(n=10\right)$. One-way ANOVA: $F_{2,27}=0.217$, p=0.806*,* $\eta^{2}=0.016$. Different uppercase letters indicate significant differences in post hoc comparisons; groups sharing the same letter are not significantly different.

Group	Mean ± SD	Median	Min–Max	Statistical Results
Manual	154.58 ± 20.56	151.76	126.91–188.63	A
Electric	159.54 ± 25.35	153.66	108.19–196.09	A
Sonic	153.88 ± 15.94	151.02	127.15–184.15	A

## Data Availability

The original contributions presented in this study are included in the article. Further inquiries can be directed to the corresponding author.
